# Identification of Senescence-Related Subtypes, the Development of a Prognosis Model, and Characterization of Immune Infiltration and Gut Microbiota in Colorectal Cancer

**DOI:** 10.3389/fmed.2022.916565

**Published:** 2022-05-26

**Authors:** Ju-Ji Dai, Yang-Yang Fu, Xi-Qiang Zhong, Wei Cen, Mao-Fei Ye, Xi-Han Chen, Yi-Fei Pan, Le-Chi Ye

**Affiliations:** ^1^Department of Colorectal and Anal Surgery, The First Affiliated Hospital of Wenzhou Medical University, Wenzhou, China; ^2^Division of Pulmonary Medicine, Key Laboratory of Heart and Lung, The First Affiliated Hospital of Wenzhou Medical University, Wenzhou, China; ^3^Department of Spinal Surgery, The Third Affiliated Hospital of Wenzhou Medical University, Wenzhou, China; ^4^Department of Urology, Shanghai Baoshan Luodian Hospital, Shanghai, China; ^5^Department of Gastroenterology, The People's Hospital of Pingyang, Wenzhou, China

**Keywords:** colorectal cancer, senescence, tumor microenvironment, gut microbiota, prognosis

## Abstract

Cellular senescence is associated with tumorigenesis, and the subtype and prognostic signatures of senescence-related genes (SRGs) in the tumor microenvironment (TME) and gut microbiota have not been fully determined. Analysis of 91 SRGs obtained from the GSEA and MSigDB, and mRNA sequencing of genes in the Gene Expression Omnibus (GEO) and The Cancer Genome Atlas (TCGA) databases enabled the identification of two distinct molecular types of colorectal cancer (CRC). Patient samples were clustered into two subtypes, with Kaplan-Meier survival analyses showing significant differences in patient survival between the two subtypes. Cluster C2 was associated with patient clinicopathological features, high immune score, high abundance of immune infiltrating cells and somewhat high abundance of bacteria. A risk model based on eight SRGs showed that a low risk score was characterized by inhibition of immune activity and was indicative of better prognosis in patients with CRC. In combination with clinical characteristics, risk score was found to be an independent prognostic predictor of survival in patients with CRC. In conclusion, the present study showed that senescence-related subtypes and a signature consisting of eight SRGs were associated with CRC patient prognosis, as well as with immune cell infiltration and gut microbiota. These findings may enable better prediction of CRC patient prognosis and facilitate individualized treatments.

## Introduction

Colorectal cancer (CRC) is the third most common cancer and the second leading cause of cancer-related deaths worldwide (1). Treatment of early stage CRC patients consists primarily of surgery and adjuvant chemotherapy, with these patients having a 5-year overall survival (OS) rate of 90% (2). Combinations of targeted drugs and chemotherapy, which are currently the first-line treatment for metastatic CRC (mCRC), can improve the efficacy of mCRC treatment and prolong patient survival (3). However, the 5-year OS rate in patients with mCRC, consisting of metastases to the liver, lungs, brain, and other organs, rate is only approximately 10% (4). Therefore, the mechanisms underlying tumor progression in patients with CRC need further study.

Cellular senescence is a type of cellular program responsible for inducing cell cycle arrest. This program is characterized by distinct phenotypic alterations, including expression of anti-proliferative molecules and activation of damage sensing signaling pathways (5). The growth arrest of proliferating cells is typically triggered by constant DNA damage response (DDR) or other types of stress signaling, such as telomere dysfunction and oncogene activation (6). Cellular senescence prevents cancer in mammals. Oncogene activation triggers an initial hyperproliferative response intrinsically related to changed DNA replication, ultimately engaging DDR pathways and inducing cellular senescence (7). Several drugs that have been shown to enhance DNA damage and induce tumor cell senescence have been utilized in the management of human cancers (8). Senescent tumor cells can alter the tumor microenvironment (TME) via the senescence-associated secretory phenotype (SASP). The SASP consists of several chemokines and cytokines that activate immune surveillance and trigger innate and adaptive immune responses, which clear senescent and proliferating tumor cells (9). To date, several risk modes have been developed to evaluate the prognostic value of genes related to the TME in CRC. Less is known, however, about the involvement of senescence-related genes (SRGs).

In the present study, SRGs in CRC were comprehensively investigated to determine the mechanisms by which senescence affects immune cell infiltration and gut microbiota in CRC. Utilizing data from the Gene Expression Omnibus (GEO) and The Cancer Genome Atlas (TCGA) databases led to the establishment of a consistent clustering and SRG-based model to evaluate the prognostic value of SRGs in CRC. The association of senescence with immune cell infiltration and gut microbiota was explored in CRC. These findings may contribute to designing comprehensive methods of treating CRC and enable targeted treatment to individual patients.

## Materials and Methods

### Data Collection

Data on the clinicopathological characteristics and mRNA expression of TCGA CRC cohorts were obtained from the UCSC database (https://xenabrowser.net/datapages/) and used as a training cohort. In addition, clinical information and mRNA sequencing results associated with the GSE39582, GSE17536, GSE17537 and GSE19072 datasets of patients with CRC were obtained from the GEO database (https://www.ncbi.nlm.nih.gov/geo/). Four GEO datasets were combined, and batch effects were eliminated by applying the “Combat” algorithm. Specimens lacking complete clinical information, including patient sex, age, survival status, duration of survival and TNM stage, were excluded. Microbial abundance profiles at the genus, order, phylum, class, and family levels were obtained from the TCMA database (https://tcma.pratt.duke.edu/). Immunohistochemistry (IHC) staining of the SRGs in CRC and normal tissues was acquired from The Human Protein Atlas (THPA) database (https://www.proteinatlas.org/).

### Consensus Clustering Analysis of SRGs

Ninety-one SRGs identified in the GSEA and MSigDB databases (GOBP_CELLULAR_SENESCENCE) (https://www.gsea-msigdb.org/gsea/index.jsp) were surveyed (Supplementary Table 1) and subjected to cluster analyses using ConsensusClusterPlus. Agglomerative pam clustering featuring a Pearson correlation distances of 1 was performed and 80% of the specimens were resampled for ten repetitions. The best quantity of clusters was measured using the empirical cumulative dispersion function (CDF) diagram.

### Evaluation of Immune Infiltration

The immune infiltrating score of all samples was calculated using R package programs CIBERSORT, ESTIMATE, and TIMER.

### Identification and Functional Analysis of Differentially Expressed Genes (DEGs)

DEGs in two clusters, defined as those with a ≥1.5-fold difference in expression and an adjusted *p*-value of < 0.05, were determined utilizing the R package “limma” (version 3.40.6). For gene set functional enrichment analyses, the Gene Ontology (GO) subset from the MSigDB (http://www.gsea-msigdb.org/gsea/downloads.jsp) and KEGG pathways from the KEGG rest API (https://www.kegg.jp/kegg/rest/keggapi.html) were mapped to the TCGA database using the R software package clusterProfiler (version 3.14.3). GSEA software (version 3.0) was obtained from the GSEA website (http://software.broadinstitute.org/gsea/index.jsp), and c2.cp.kegg.v7.4.symbols.gmt was downloaded from the MSigDB Website (http://www.gsea-msigdb.org/gsea/downloads.jsp). Associated paths and molecular mechanisms were evaluated based on phenotypes and gene expression profiles. Gene sets were defined as ranging from 5 to 5,000 genes with 1,000 replicates. An FDR < 0.25 and a *P*-value < 0.05 were regarded as statistically significant.

### Identification and Verification of Risk Modes

Using the “glmnet” package in R, least absolute shrinkage and selection operator (LASSO) Cox regression analyses were performed to minimize over-fitting risks. An optimal risk model was determined by 10-fold cross-verification using the equation,

Risk score = ∑(gene Expression^*^ gene coefficient).

Patients were classified into low-risk and high-risk groups based their median risk score. Principal component analyses (PCA) were performed using the “stats (version 3.6.0)” package in R. The z-scores of the expression profiles were determined, and a reduced dimensionality matrix was obtained using the prcomp function.

### Establishment and Assessment of a Predictive Nomogram

Using the “rms” package in R and the factors survival time, survival status, and four clinical characteristics, Cox proportional analyses were performed to develop a nomogram predictive of OS. Nomograms were assessed by time-dependent receiver operator characteristic (ROC) curves for one, three, and five-year survival. Accuracy was verified by calibration plots.

### Statistical Analyses

All statistical analyses were performed using R version 4.1.0 software. Continuous variables were reported as mean ± SD as appropriate and compared by Student's *t*-tests. Survival was analyzed using the Kaplan-Meier method and compared by log-rank tests. Time-dependent ROC analyses were performed using the “survivalROC” package in R software to evaluate factors associated with OS. A *p*-value < 0.05 was defined as statistically significant.

## Results

### Identification of Senescence Subtypes in CRC

**Figure 1:**
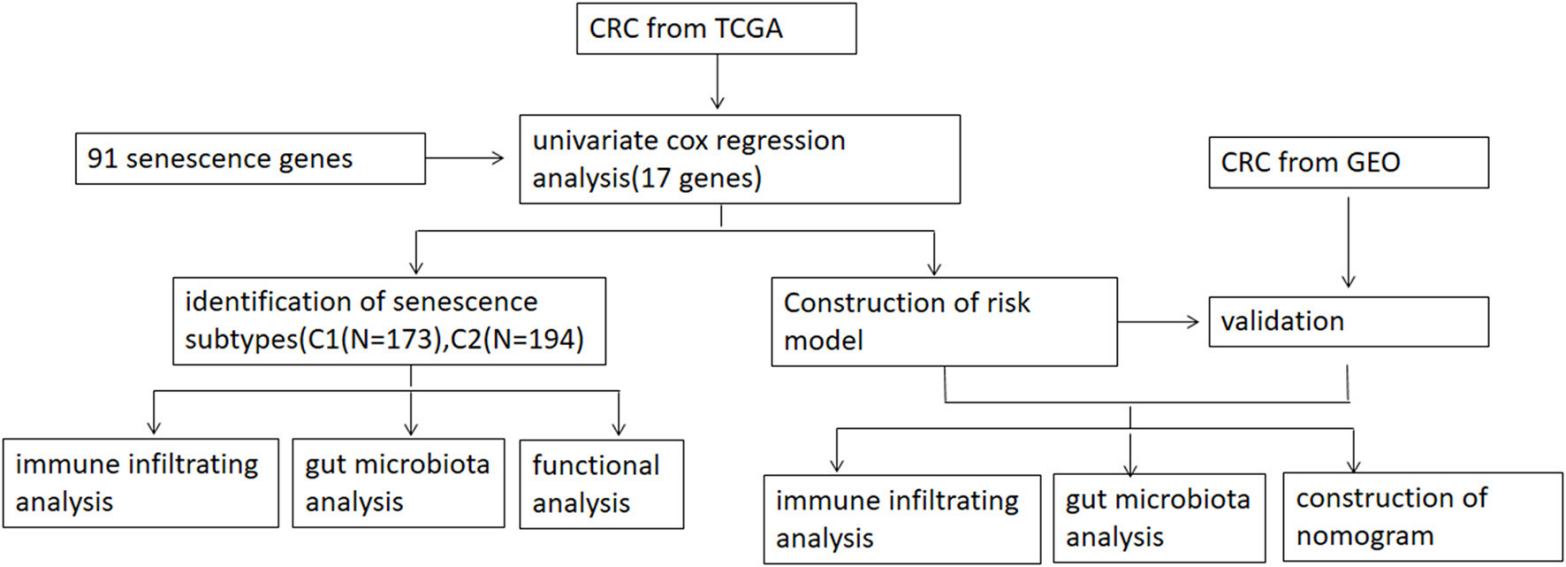
Workflow of the analytic process.

**Figure 2:**
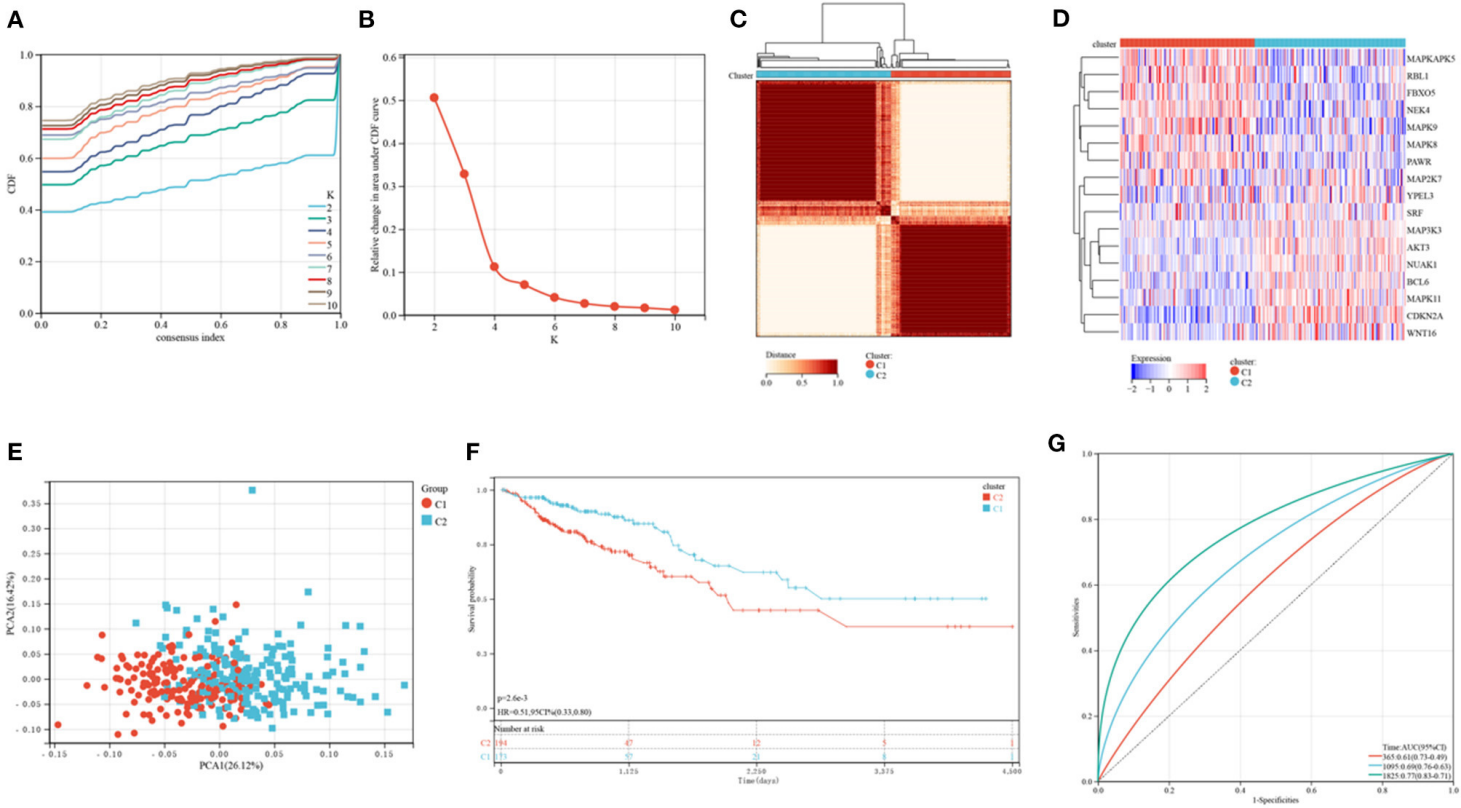
Consensus clustering of CRC subtypes based on SRGs. **(A)** CDF plots of the consensus score (*k* = 2–10). **(B)** Relative alterations in the regions under the CDF plot (*k* = 2–10). **(C)** Consensus matrix heatmap identifying two clusters (*k* = 2) and their related regions. **(D)** Expression of SRGs in the two distinctive subtypes. **(E)** PCA analyses showing marked differences in the transcriptomes of the two subtypes. **(F)** Kaplan-Meier analyses of OS of patients in the two subtypes. **(G)** ROC for predicting the specificity and sensitivity of 1, 3, and 5-year survival of patients in the two subtypes.

Figure 1 shows a map of the process of the present work. Using the consensus clustering method, patients with CRC patients in the training cohorts were divided into subgroups based on 17 genes prognostic of senescence identified on univariable Cox analyses (Supplementary Table 1). Optimal cluster stability was defined as K = 2 (Figures 2A–C and Supplementary Figure 1). Of the 367 patients evaluated, 173 were assigned to cluster C1 and 194 to cluster C2. A heatmap comparing the expression of SRGs in these two clusters showed significant differences in expression in clusters C1 and C2 (Figure 2D). Furthermore, comparisons of the clinicopathological features of the two subtypes revealed that cluster C1 was significantly related to more additional pharmaceutical therapy (*p* < 0.01), more additional radiation therapy (*p* < 0.05), higher pathologic T (*p* < 0.01), higher pathologic N (*p* < 0.001), higher pathologic stage (*p* < 0.001) and more venous invasion (*p* < 0.001) compared to those in cluster C2 (Supplementary Table 2). PCA analyses showed marked differences in the senescence transcription profiles of the two clusters (Figure 2E), whereas Kaplan-Meier analysis showed that OS was significantly longer in cluster C1 than in cluster C2 (*P* = 0.0026; Figure 2F). ROC analysis showed that the 1, 3, and 5-year OS of patients based on SRG scores were yielded AUCs of 0.61, 0.69, and 0.77, respectively (Figure 2G). These findings showed that SRGs could divide CRC patients into two molecular subtypes with significant differences in OS.

### Characteristics of the Immune States in the Two Clusters

**Figure 3:**
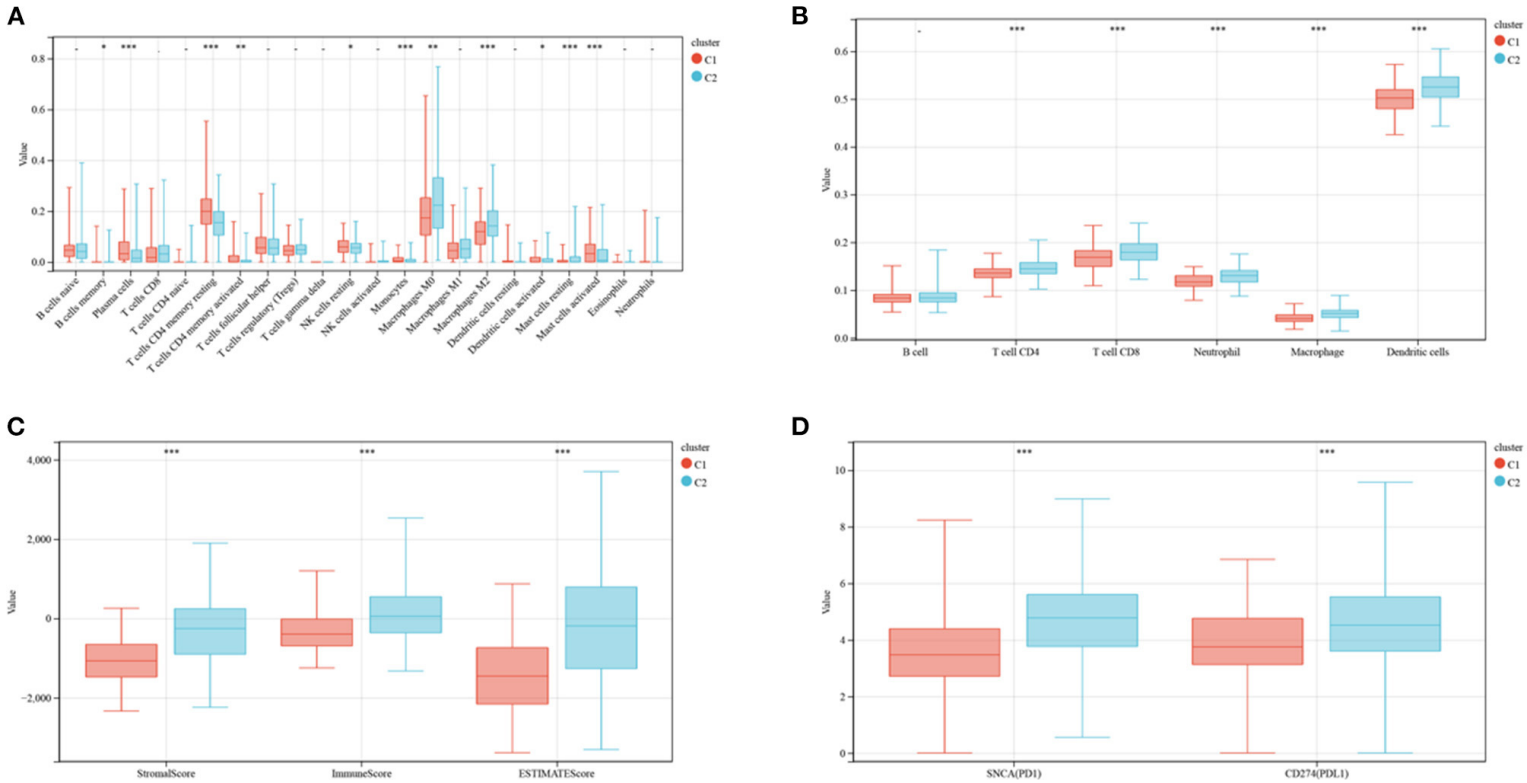
Evaluation of immune infiltration and checkpoints in the two subtypes. **(A)** CIBERSORT analysis of the abundance of 22 immune cells infiltrating tumors of the two subtypes. **(B)** TIMER analysis of the abundance of six immune cells infiltrating tumors of the two subtypes. **(C)** Stromal, immune and ESTIMATE scores of patients in the two subtypes. **(D)** Expression of immune checkpoints by tumors in the two subtypes.

The enrichment of 22 human immune cell subsets in each CRC sample was evaluated using CIBERSORT analysis. Patients in clusters C1 and C2 showed marked differences in the infiltration of most immune cells (Figure 3A). The populations of infiltrating monocytes, plasma cells, resting natural killer (NK) cells, resting CD4 memory T cells, activated dendritic cells (DCs), and activated mast cells were significantly higher in cluster C1 than in cluster C2, whereas the populations of infiltrating resting mast cells, M0 and M2 macrophages, and memory B cells were significantly lower in C1 than in C2 reduced. TIMER analysis (Figure 3B) showed that the numbers of DCs (*P* < 0.001), CD8 T cells (*P* < 0.001), CD4 T cells (*P* < 0.001), neutrophils (*P* < 0.001), and macrophages (*P* < 0.001) were significantly higher in cluster C2 than in cluster C1, but there were no differences in B cell population. ESTIMATE score (P <0.001), immune score (P <0.001) and stromal score (P <0.001) were all significantly higher in cluster C2 than in cluster C1 (Figure 3C). Similarly, analyses of two key immune checkpoints showed that the levels of expression of *PD1* and *PD-L1* were higher in cluster C2 than in C1 (Figure 3D).

### Differential Microbiome Profiling of CRC in the Two Clusters

**Figure 4:**
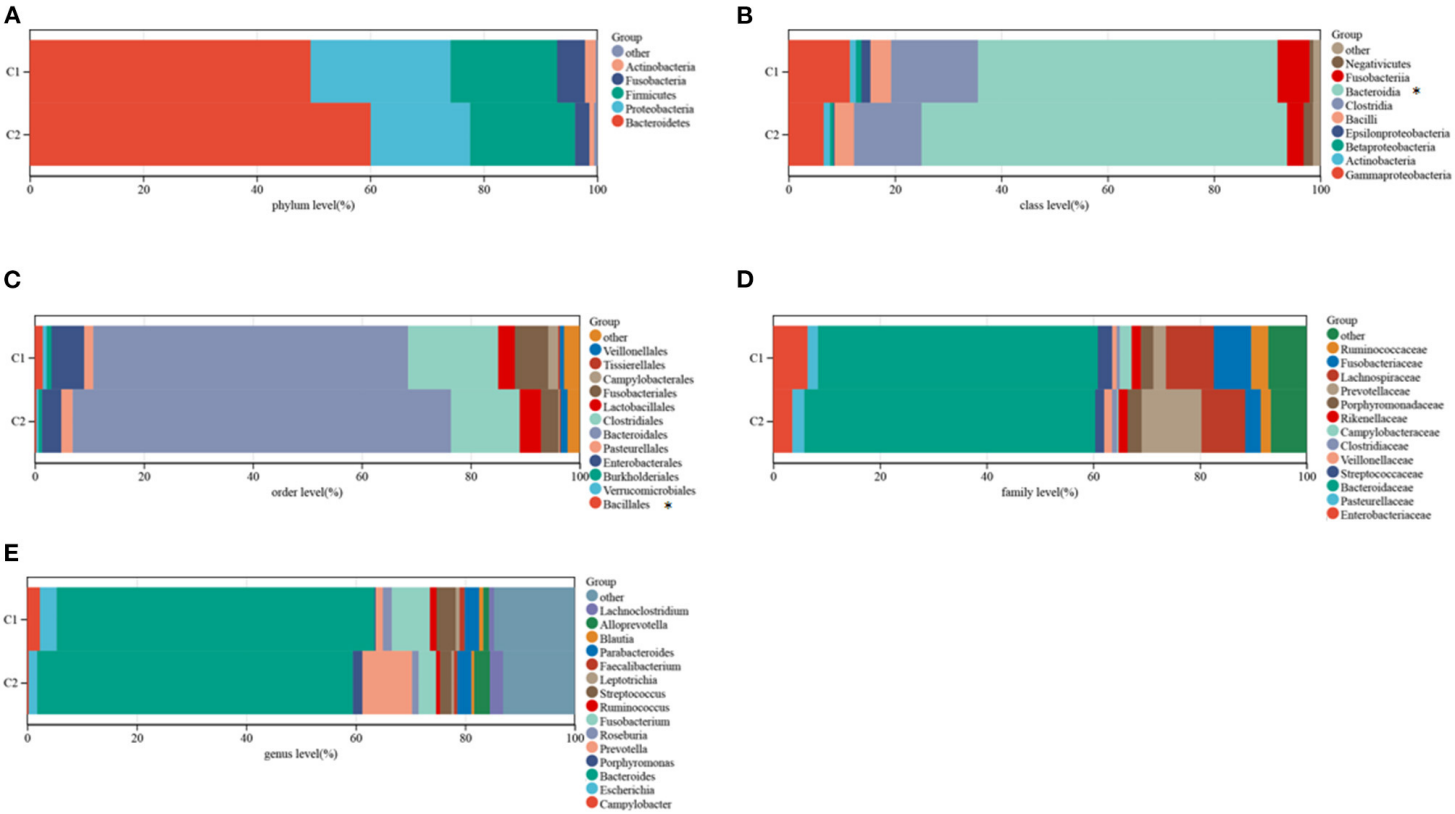
Composition of microbiota in the two subtypes at the phylum **(A)**, class **(B)**, order **(C)**, family **(D)**, and genus **(E)** levels.

The TCMA database was searched to identify microbial samples in all CRC samples. A total of 11 phyla, 22 classes, 38 orders, 76 families, and 221 genera of microbes were surveyed (Supplementary Table 3). Most CRC samples showed enrichment of 5 phyla, 9 classes, 12 orders, 15 genera, and 13 families of microbes (Figures 4A–E). At the class level, the intra-tissue microbiota were dominated by the classes *Bacteroidia* (present in 55.4 and 67.5% of samples in clusters C1 and C2, respectively), *Clostridia* (present in 16.0 and 12.5% of samples in clusters C1 and C2, respectively), and *Gammaproteobacteria* (present in 11.4 and 6.5% of samples in clusters C1 and C2, respectively) (Figure 4E). Although the relative abundance of class *Bacteroidia* was lower in cluster C1 than in cluster C2 group, the relative abundance of the order *Bacillales* was higher in cluster C1 (Figure 4E).

### DEGs and Functional Analysis

**Figure 5:**
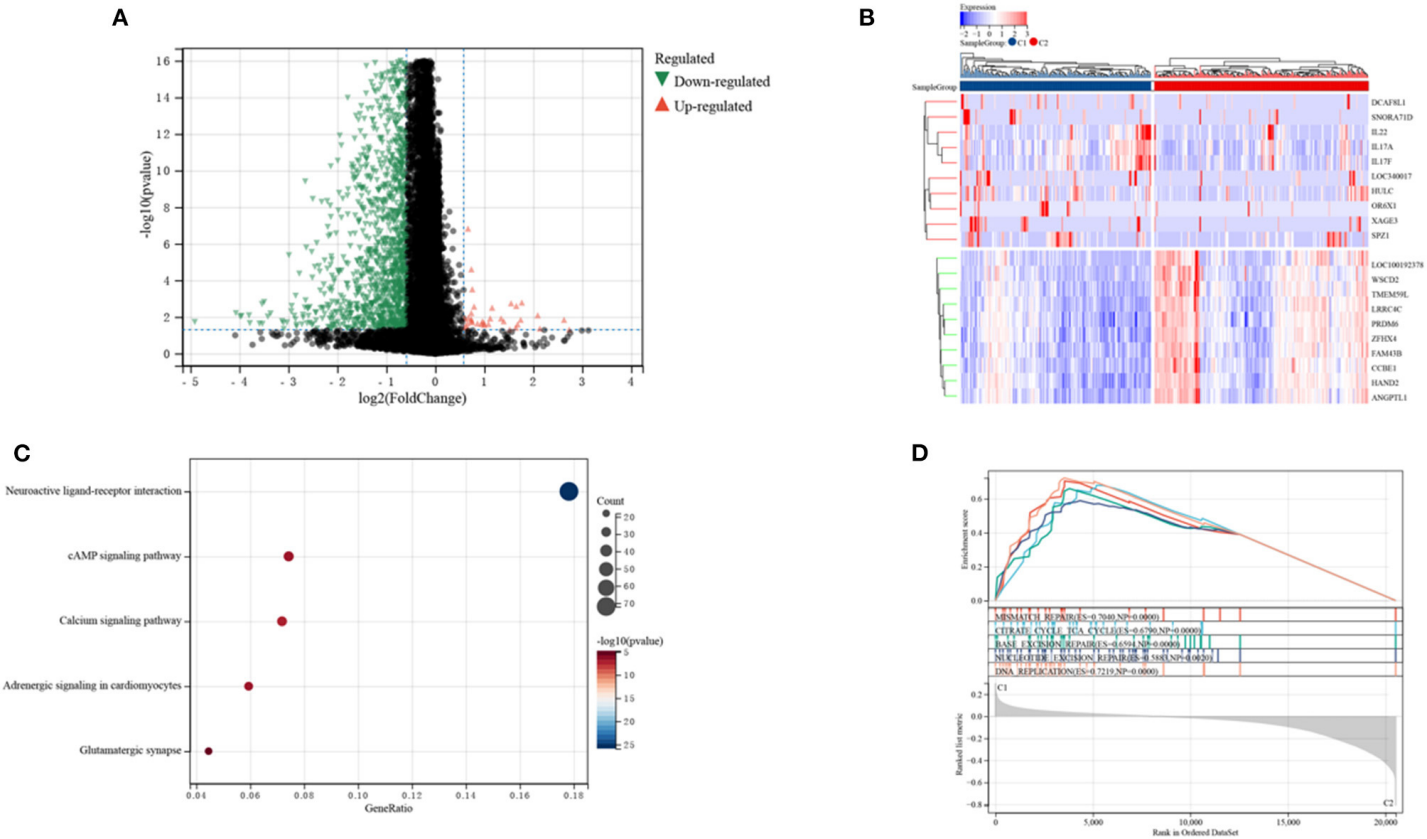
Analyses of differentially expressed genes (DEGs) and of gene function in the two subtypes. **(A)** Volcano plot showing DEGs by the two subtypes. **(B)** Heatmap of the top 10 DEGs in each of the two subtypes. **(C)** KEGG enrichment analyses of DEGs in the two subtypes. **(D)** GSEA analyses of SRGs in the two subtypes.

The DEGs in the two clusters were subjected to functional analysis to explore potential signaling mechanisms. Cluster C1 showed upregulation of 37 genes and downregulation of 1,281 genes compared with cluster C2 (Figure 5A), with a heatmap showing the top 10 differentially expressed genes in the two clusters (Figure 5B). GO enrichment analyses showed that senescence was associated with neural signal transmission and transport, including G protein-coupled receptor signaling pathways, synapses, and receptor regulatory activity (Supplementary Figure 2). KEGG enrichment analyses also identified several signaling pathways associated with neural signaling, including neuroactive ligand-receptor interaction, cAMP signaling, calcium signaling, adrenergic signaling in cardiomyocytes, and glutamatergic synapses (Figure 5C). The link between enriched pathways and prognosis in patients with CRC was evaluated by GSEA analysis of differences in expression of pathways in the two patient clusters. GSEA analyses showed that mismatch repair, citrate cycle, TCA cycle, DNA replication, nucleotide excision repairs, base excision repairs and other signaling pathways related to cell replication were more highly expressed in cluster C1 than in cluster C2 (Figure 5D). Taken together, these finding suggest that SRGs are associated with cell replication and neural signal transmission, which may be related to better prognosis in patients with CRC.

### Construction of the Prognosis Model

**Figure 6:**
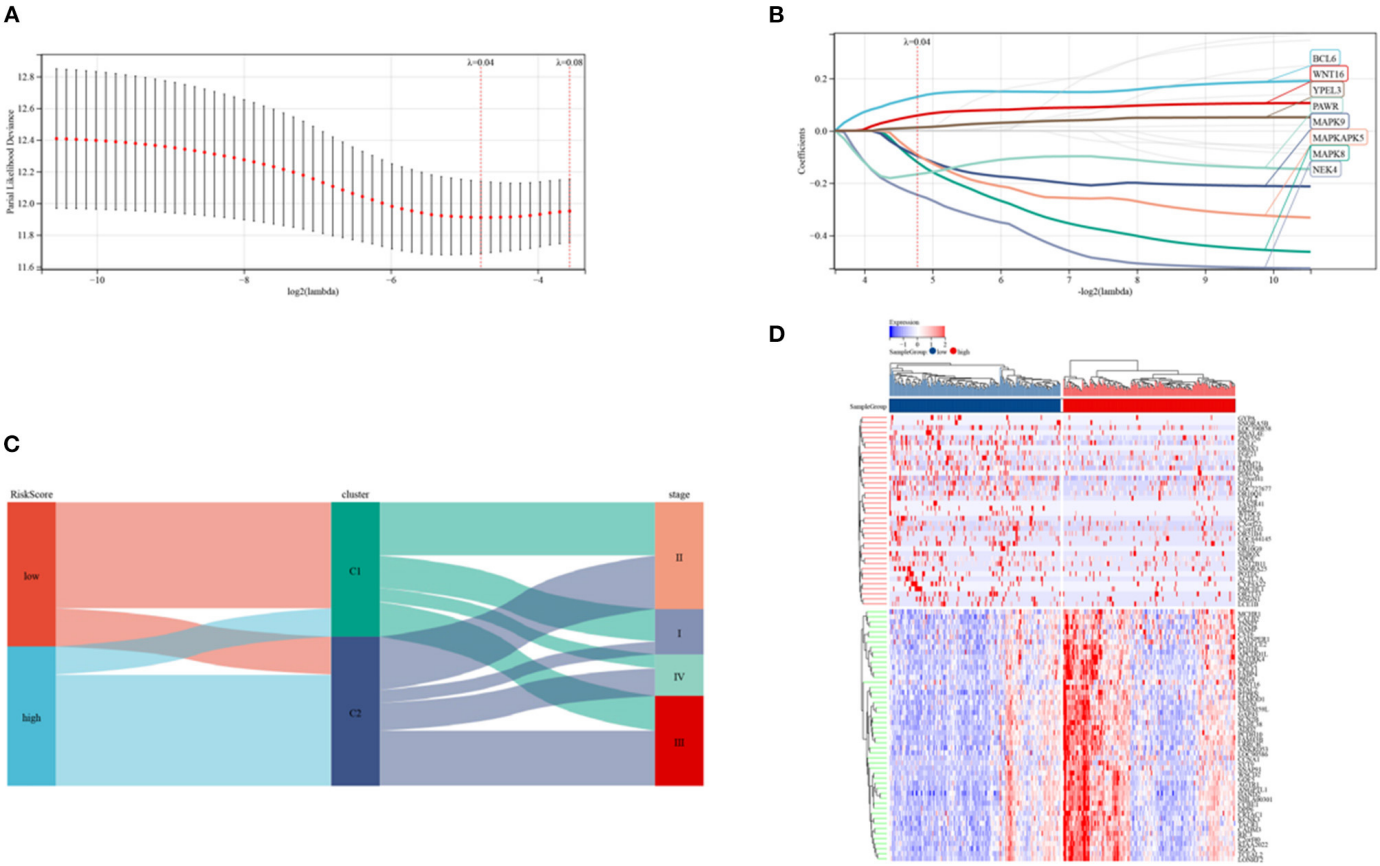
Establishment of a risk model in the TCGA cohort. **(A,B)** LASSO regression analyses and partial family deviance of the prognostic genes. **(C)** Alluvial plot of sub-type dispersion in groups with various SRG risk scores, clusters and CRC stages. **(D)** Heatmap of the top 50 DEGs in the low and high-risk groups.

Risk signature models were developed to evaluate the prognostic predictive ability of SRGs in CRC. Screening of potential genes by LASSO analysis (Figures 6A,B) to establish the risk mode identified eight genes with optimal lambda values (risk score = 0.129607980597333 ^*^ *BCL6*- 0.119970227463924 ^*^ *MAPK8*- 0.0962359783869445 ^*^ *MAPK9*- 0.0911883032223032 ^*^ *MAPKAPK5*- 0.245073218298237 ^*^ *NEK4*- 0.166348735320059 ^*^ *PAWR* + 0.0583076641122957 ^*^ *WNT16* + 0.012344436894066 ^*^ *YPEL3*). This risk mode divided CRC patients into low- and high-risk groups. Patients were categorized by senescence subtypes, risk groups and clinical stage (Figure 6C), with Figure 6D showing the expression of differentially expressed genes in the two risk groups.

**Figure 7:**
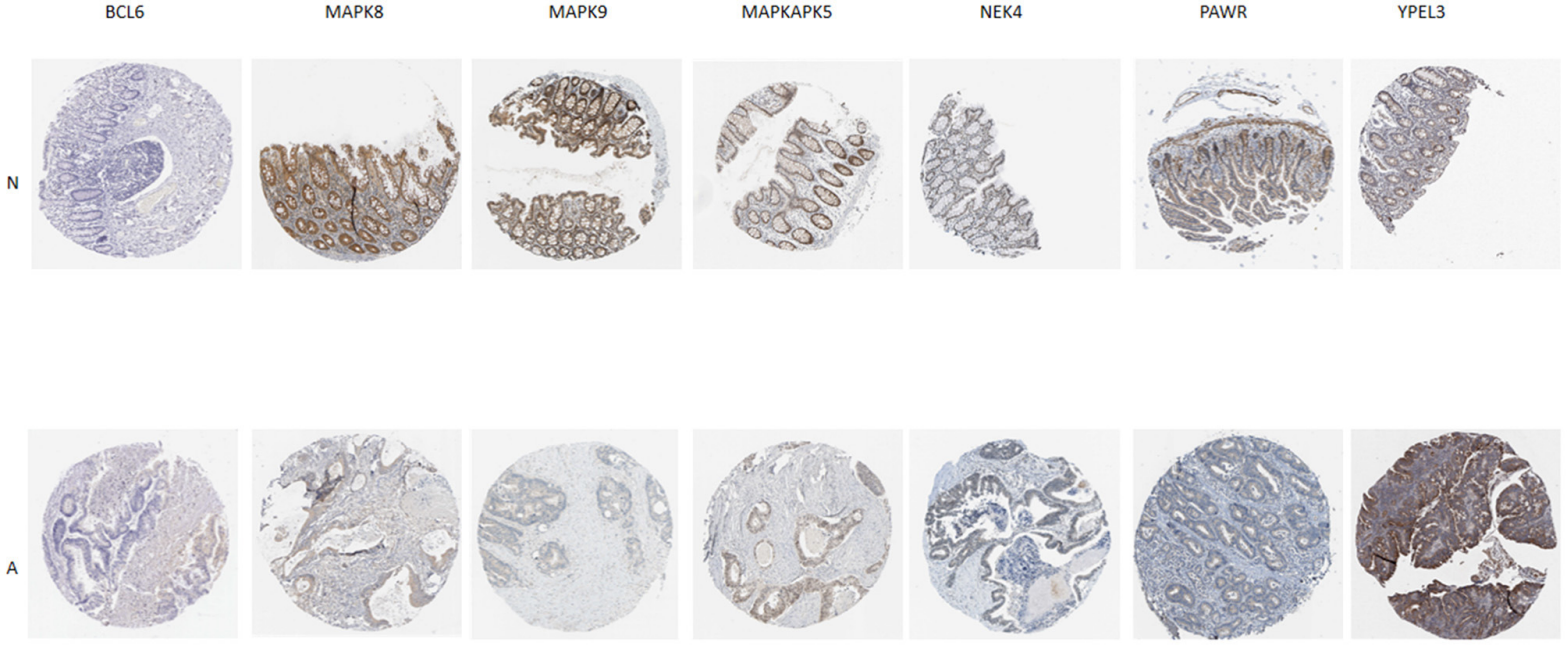
Verification of SRG expressions in normal and tumor tissue by immunohistochemistry staining based on The Human Protein Atlas (THPA) database. N represent normal tissue, A represent CRC tissue.

Additionally, IHC staining of SRGs in CRC and normal tissues was acquired from THPA database. BCL6 and YPEL3 were upregulated in CRC, while MAPK8, MAPK9, MAPKAPK5, NEK4, and PAWR were downregulated in CRC (Figure 7). However, the IHC images of WNT16 were not found.

**Figure 8:**
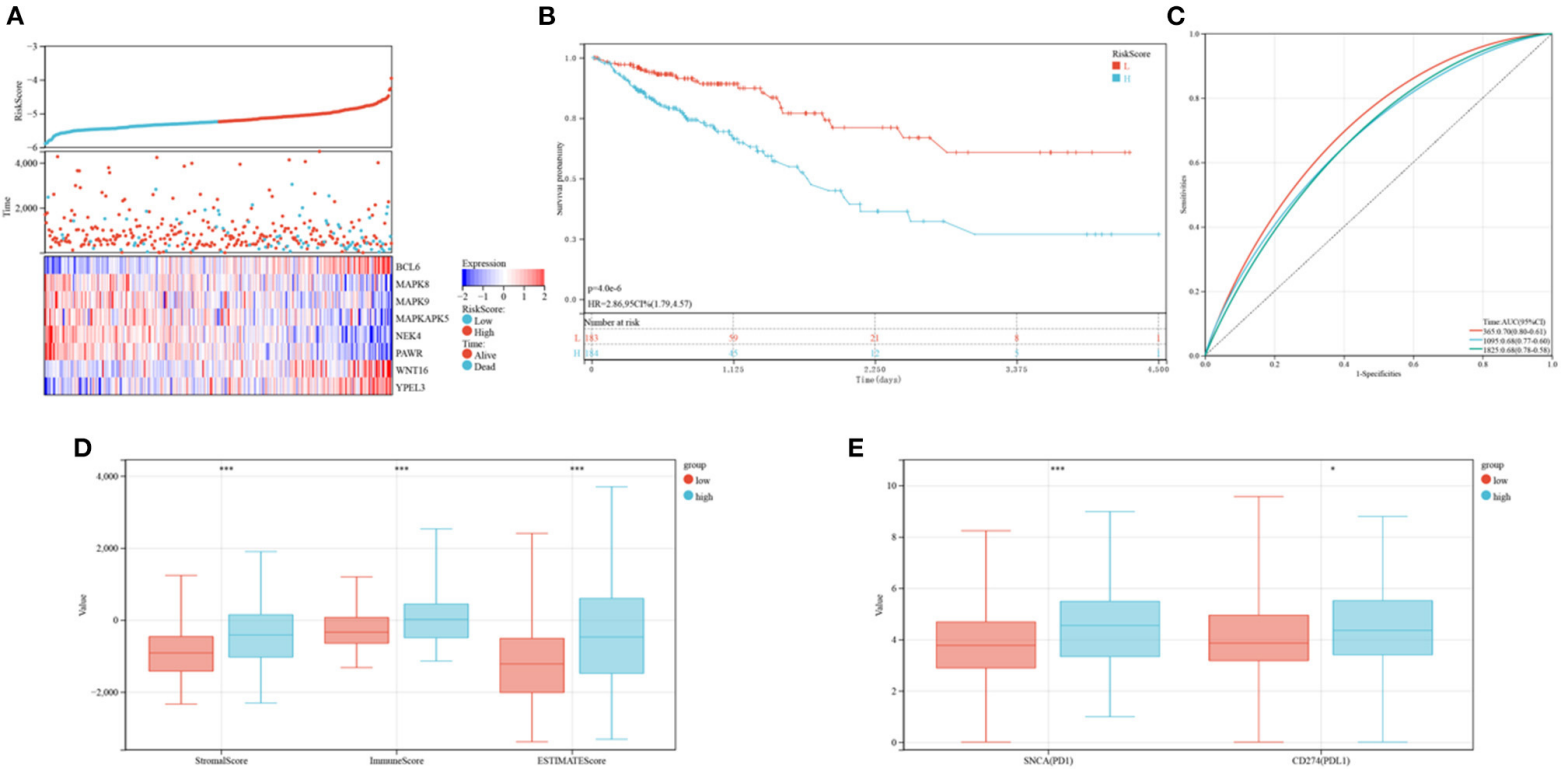
Prognoses and immune characteristics of the low and high-risk groups in the TCGA cohorts. **(A)** Distribution of risk scores and survival status of CRC patients with SRG expression. **(B)** Kaplan-Meier analysis of OS in the low and high-risk groups. **(C)** ROC plots predicting the specificity and sensitivity of 1-, 3-, and 5-year survival in the low and high-risk groups. **(D)** Comparison of stromal, immune and ESTIMATE scores in the low and high-risk groups. **(E)** Expression of immune checkpoints in the low and high-risk groups.

The levels of expression of *BCL6, WNT16*, and *YPEL3* were higher in the high-risk group than in the low-risk group, whereas the levels of expression of *MAPK8, MAPK9, MAPKAPK5, NEK4*, and *PAWR* were higher in the low-risk group (Figure 8A). OS was significantly longer in the low-risk than in the high-risk group (Figure 8B). Time-dependent ROC analyses showed that risk mode was accurately predictive of OS over 5 years, with the AUCs of ROC curves at 1, 3, and 5 years being 0.70, 0.68, and 0.68, respectively (Figure 8C). Use of the ESTIMATE algorithm in the two groups showed that ESTIMATE score (*P* < 0.001), immune score (*P* < 0.001) and stromal score (*P* < 0.001) were significantly higher in high-risk than in low-risk patients (Figure 8D). Similarly, the levels of expression of two key immune checkpoints, *PD-L1* and *PD1*, were significantly higher in the high-risk group (Figure 8E).

**Figure 9:**
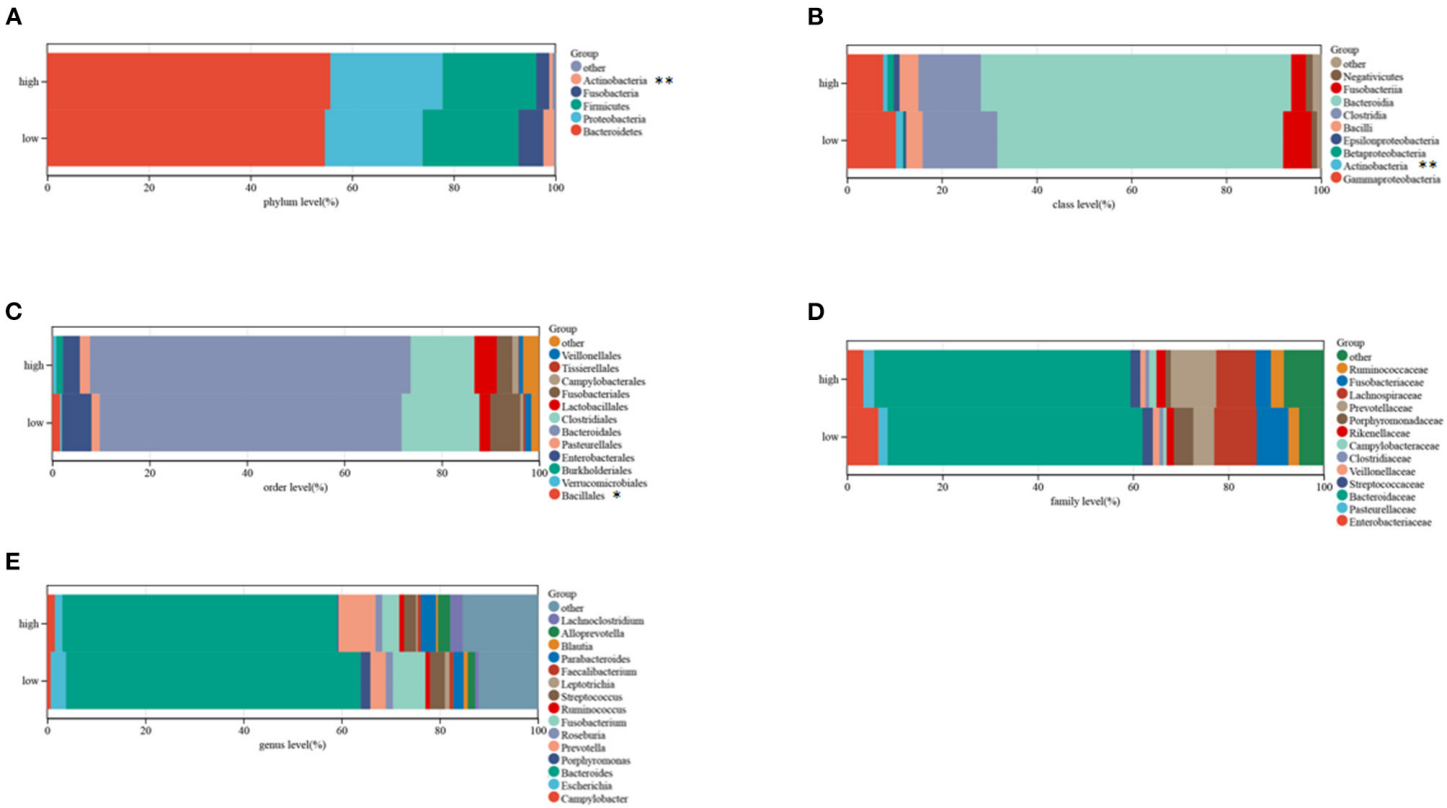
Composition of microbiota in the low and high-risk groups at the phylum **(A)**, class **(B)**, order **(C)**, family **(D)**, and genus **(E)** levels, separately.

Microbiome profiling of CRC patients showed that the relative abundances of the phylum *Actinobacteria*, the *Bacillales* and the class *Actinobacteria* were lower in high-risk than in low-risk patients (Figures 9A–E). These outcomes suggest that this risk model can anticipate the prognosis of CRC patients and is related to immune status and gut microbiota in patients with CRC.

### Verification of the Prognosis Model in the GEO Database

**Figure 10:**
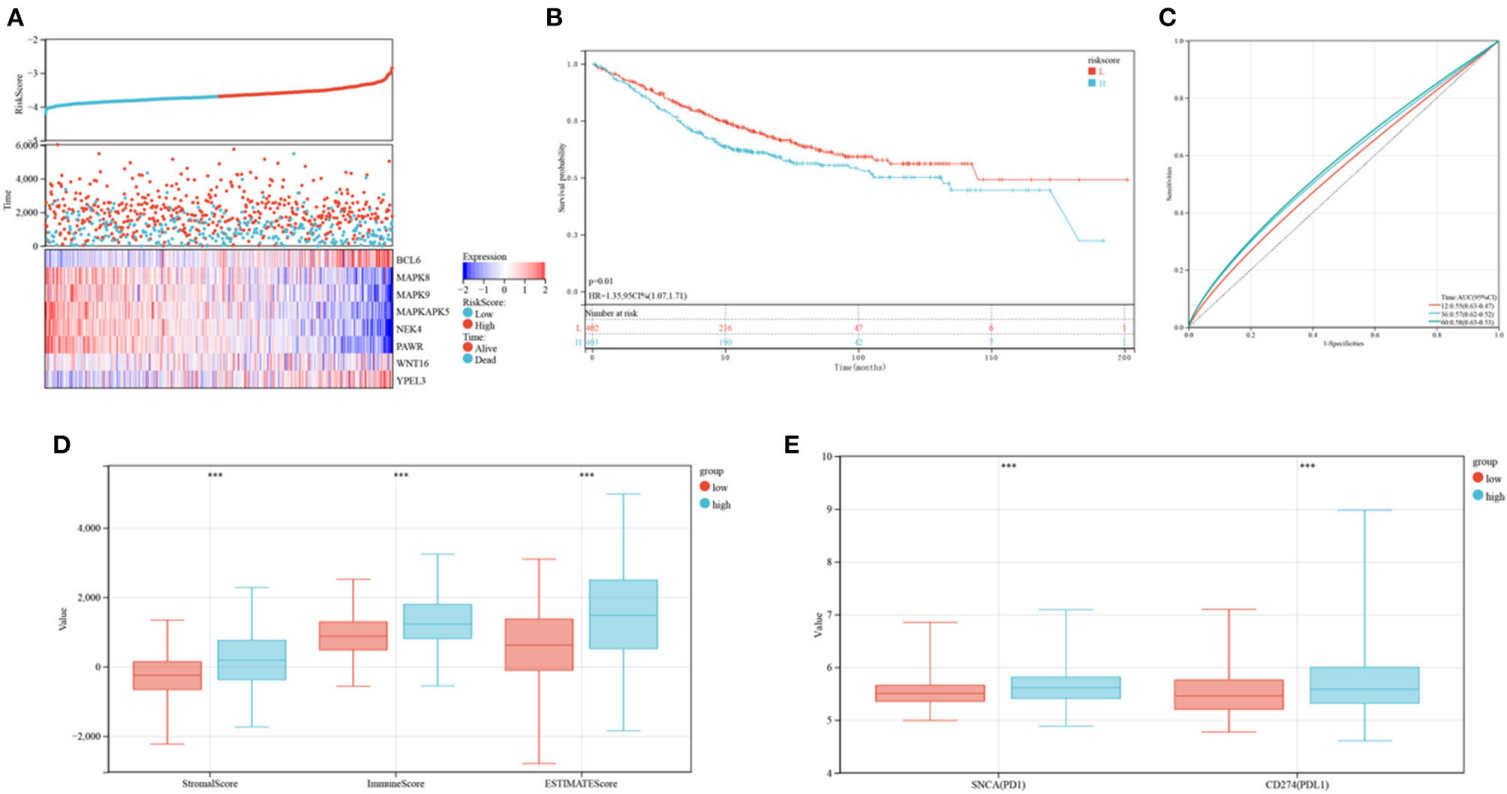
Verification of the established risk mode in GEO cohorts. **(A)** Distribution of risk scores and survival status of CRC patients as a function of SRG expression. **(B)** Kaplan-Meier analysis of OS in the low and high-risk groups. **(C)** ROC plots predicting the specificity and sensitivity of 1, 3, and 5-year survival in the low and high-risk groups. **(D)** Comparison of stromal, immune and ESTIMATE scores in the low and high-risk groups. **(E)** Expression of immune checkpoints in the low and high-risk groups.

The developed prognostic risk score model was further validated in the validation cohort. CRC patients in the validation cohorts were stratified as low or high-risk, and their levels of expression of the eight candidate genes, the risk scores and survival events were compared (Figure 10A). Survival analyses showed that patients in the high-risk group had poorer prognoses (*P* = 0.01; Figure 10B), whereas ROC analyses found that the risk model was optimal at determining 1, 3, and 5-year OS (Figure 10C). The association between risk models and the immune microenvironment were also determined. Same to findings in the training cohorts, the levels of *PD1* and *PD-L1*, as well as ESTIMATE score (P < 0.001), immune score (*P* < 0.001), and stromal score (*P* < 0.001), were higher in the high risk than in the low-risk group (Figures 10D,E). These outcomes indicated that the developed risk model was related to the immune microenvironment and prognosis in CRC patients in the validation cohort.

### Independence of Prognostic Signature and Development of a Predictive Nomogram

**Figure 11:**
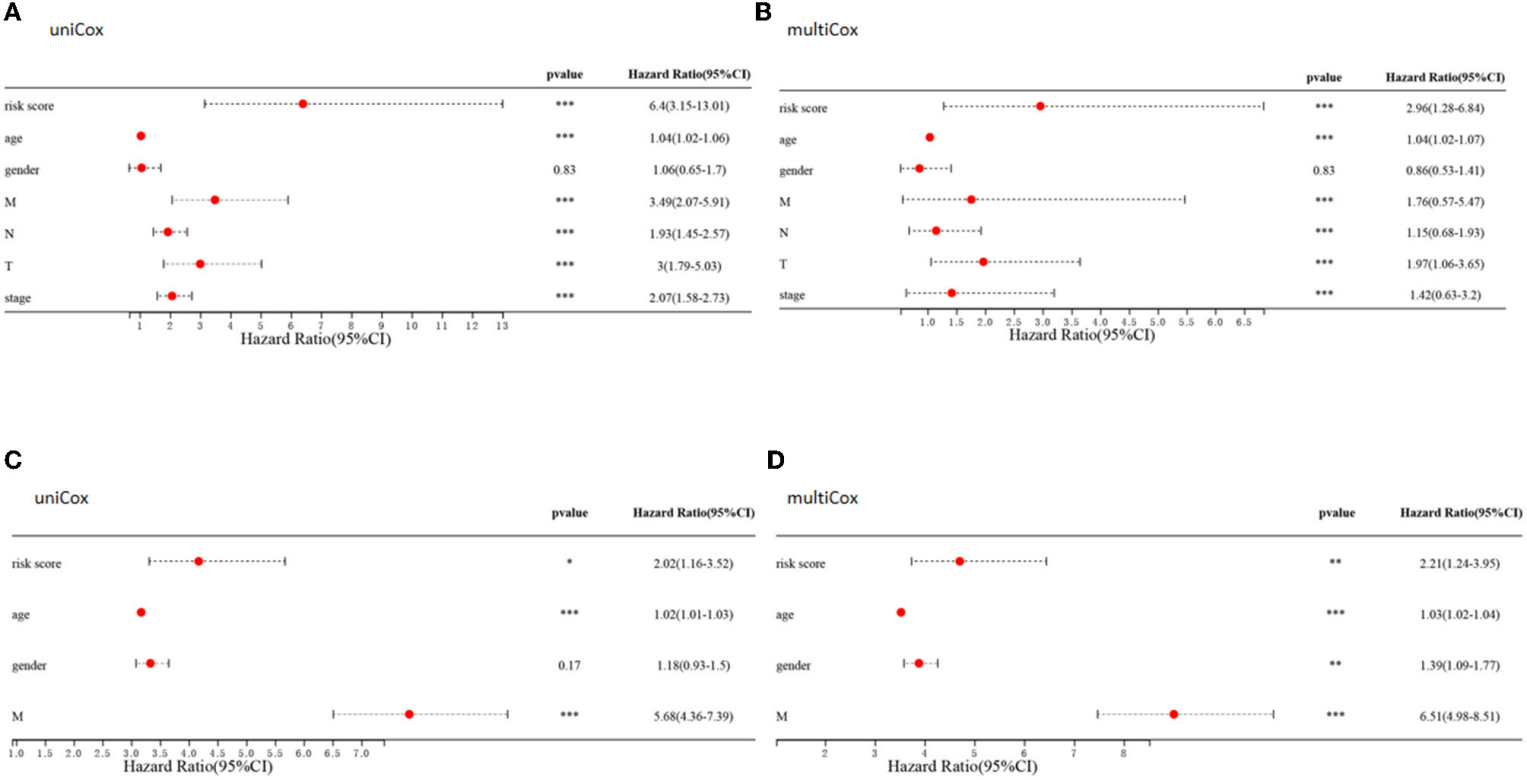
Clinical value of risk signature. **(A,C)** Forest plots of univariate Cox regression analyses for risk scores and clinical characteristics of patients in the TCGA **(A)** and GEO **(C)** cohort. **(B,D)** Forest plots of multivariate Cox regression analyses for risk scores and clinical characteristics in the TCGA **(B)** and GEO **(D)** cohorts.

Comparisons of the clinicopathological features of the low and high-risk groups revealed that high-risk groups was significantly related to more additional pharmaceutical therapy (*p* < 0.01), more additional radiation therapy (*p* < 0.05), higher recurrence risk (*p* < 0.05), more non-nodal tumor deposits (*p* < 0.01), higher metastasis risk (*p* < 0.01), higher pathologic T (*p* < 0.001), higher pathologic N (*p* < 0.001), higher pathologic stage (*p* < 0.001) and more venous invasion (*p* < 0.001) compared to those in low-risk groups (Supplementary Table 2). Furthermore, univariate and multivariate Cox regression analyses were performed to determine whether the predictive value of the prognostic model was independent of other conventional clinical features. Factors independently associated with OS in the training cohort included tumor (*P* < 0.001, hazard ratio HR = 1.97), node (*P* < 0.001, HR=1.15), and metastasis (*P* < 0.001, HR = 1.76) scores, stage (*P* < 0.001, HR = 1.42), patient age (*P* < 0.001, HR = 1.04) and risk score (*P* < 0.001, HR=2.96) (Figures 11A,B). Similarly, univariate and multivariate Cox regression analyses of the GEO database showed that patient age (P < 0.001, HR=1.03), sex (*P* < 0.01, HR = 1.39), metastasis (P < 0.001, HR=6.51) and the constructed risk model (*P* < 0.01, HR = 2.21) were independently predictive of prognosis in patients with CRC (Figures 11C,D).

**Figure 12:**
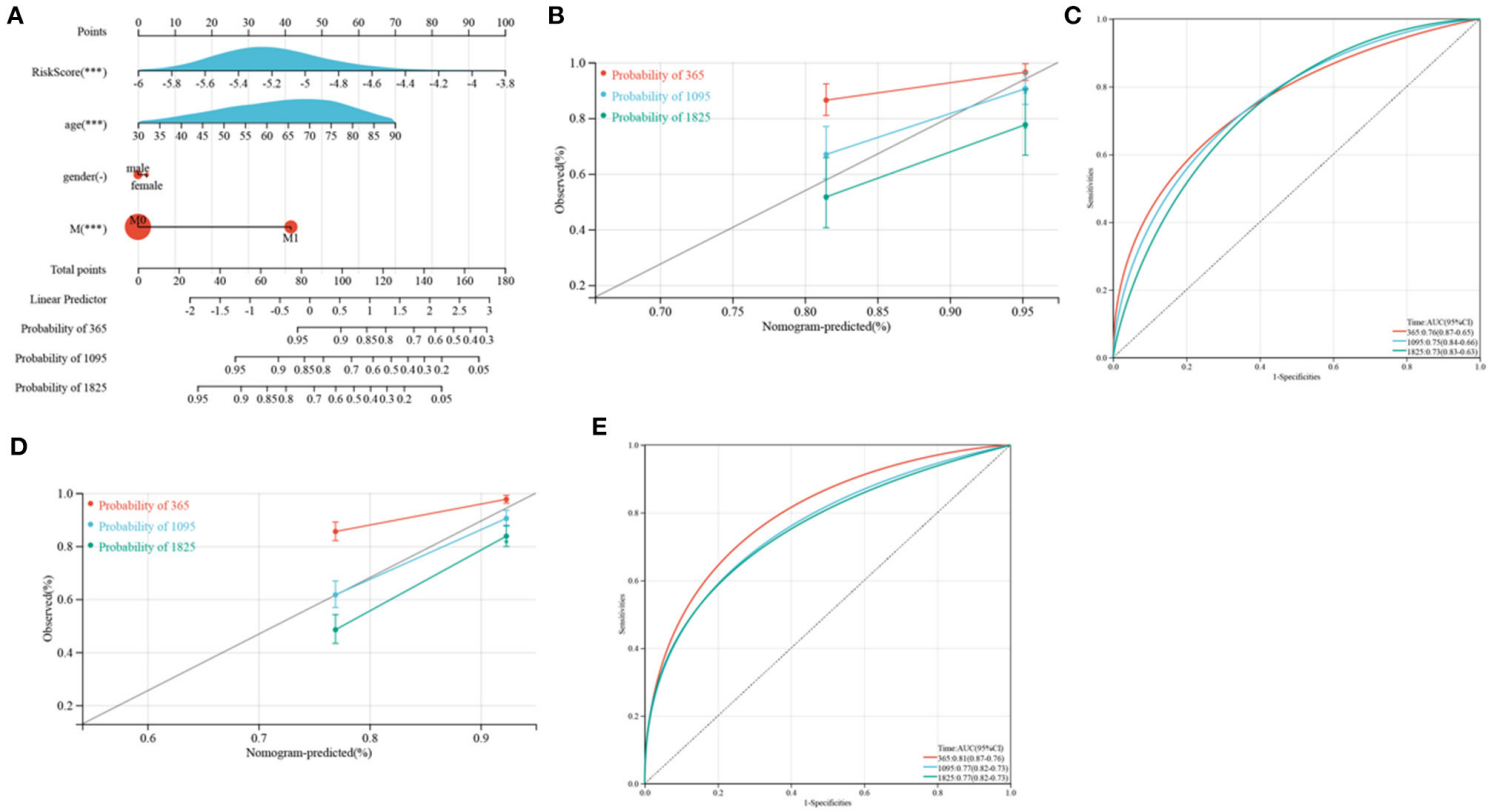
Development and verification of a nomogram. **(A)** Nomogram for anticipating the 1, 3, and 5-year OS of CRC patients in TCGA cohorts. **(B,D)** Calibration plots of the nomogram to anticipate 1, 3, and 5-year OS in the TCGA **(B)** and GEO **(D)** cohorts. **(C,E)** ROC plots predicting the 1, 3, and 5-year survival in the TCGA **(C)** and GEO **(E)** cohorts.

Because of the relatively low clinical utility of SRGs scores in predicting OS in patients with CRC, a nomogram integrating SRG scores and clinicopathological parameters was developed to predict 1, 3, and 5-year OS rates (Figure 12A). Calibration diagrams indicated that, compared with an ideal model, the nomogram had similar properties in the training and verification cohorts (Figures 12B,D). The AUCs on the nomogram model were highly precise in determining 1, 3, and 5-year OS in the training and verification cohorts (Figures 12C,E). These outcomes indicated that the nomogram may be used to accurately predict prognosis in patients with CRC.

## Discussion

Despite improvements in treat modalities, including surgical resection, targeted drug treatment and chemotherapy, the prognosis of patients with CRC is still poor. Efficient risk stratification methods and individualized treatment are necessary to improve outcomes. The present study showed that CRCs could be divided into two distinct molecular subtypes based on 17 SRGs. In comparison with patients in cluster C1, patients in cluster C2 had poorer OS and lower pathologic stage. Clusters C1 and C2 differed significantly in their infiltration by the gut flora *Bacteroidia* and by immune cells, including DCs, macrophages, memory B cells, resting NK cells, CD8 T cells, and CD4 T cells. Cell replication and neural signal transmission pathways also differed in the two clusters. These findings enabled the construction of an effective prognostic SRG risk signature, which was significantly related to senescence clusters, and showed ability to predict patient survival. Compared with patients in the low-risk group, patients in the high-risk group were characterized by poorer OS, greater tumor infiltration by immune cells and lower relative abundances of *Bacillales* and *Actinobacteria*. Integration of risk scores and clinical characteristics led to the development of a quantitative nomogram, further improving the properties and facilitating the utilization of SRG risk scores. The risk model can be adopted to predict the prognoses of patients with CRC, and may help better understand the molecular mechanisms associated with this disease.

Cellular senescence has been defined as the irreversible arrest of cell proliferation in response to oncogenic stress (10). Senescent cells can secrete many proteases, growth factors, chemokines, and pro-inflammatory cytokines, termed the SASP (11). Through the SASP, senescent cells induce paracrine senescence in neighbor cells, thereby constituting a barrier against tumor development. Because cells undergoing senescence are in constant cell cycle arrest, senescence is broadly regarded to protection against cancer by two major tumor inhibitor pathways, the ARF/p53 and the INK4a/RB paths (12, 13). The present study identified a signature involving eight SRGs, *BCL6 transcription repressor (BCL6), mitogen-activated protein kinase 8 (MAPK8), mitogen-activated protein kinase 9 (MAPK9), MAPK activated protein kinase 5 (MAPKAPK5), NIMA related kinase 4 (NEK4), pro-apoptotic WT1 regulator (PAWR), Wnt family member 16 (WNT16)*, and *yippee like 3 (YPEL3)*, predictive of the prognosis of CRC. *BCL6* was initially identified as an oncogene in B-cell lymphomas, with this gene thought to drive the malignant phenotype by repressing proliferation and DNA damage checkpoints and blocking B-cell terminal differentiation (14). In the present study, *BCL6* was found to be positively related to higher risk scores. Similarly, increased expression of BCL6 was found to be associated with the development of human CRC (15). *BCL6* was shown to participate in the regulation of Treg cellular immune responses in colorectal tumorigenesis and may be explored a therapeutic target of anti-tumor immunity (16). MAPKs have been classified as conventional and atypical MAPKs and have been shown to include ERK5, c-Jun N-terminal kinases 1-3 (JNK1-3), p38 MAPK, and extracellular signal-regulated kinases 1 and 2 (ERK1/2). *MAPK8* and *MAPK9*, also called *JNK1* and *JNK2*, respectively, are members of the *JNK* family, with JNK1 inducing cell death and JNK2 inducing cell survival (17). JNK1 inhibition sensitizes CRC cells to oxaliplatin (18), and JNK was found to induce survival-promoting autophagy, resulting in resistance to 5-FU in colon cancer cells expressing mutant *p53* (19). MAPKAPK5 can be activated by traditional (p38) and atypical (ERK3 and ERK4) MAPKs (20) and can prevent ERK3 from inhibiting cell cycle progression and controlling cell proliferation by stimulating the transcriptional activity of *p53* (21). *MAPKAPK5* is therefore a tumor inhibitor that disrupts the negative feedback loop with *myc* during CRC tumorigenesis (22). The present study found that *MAPKAPK5* was negatively related to increased risk score, providing further evidence that *MAPKAPK5* acts as a tumor suppressor. *NEK4* is a member of the *NEK* family that is overexpressed in CRC (23). NEK4 functions as a DNA damage response protein and in the stabilization of primary cilia and microtubules (24). Suppression of *NEK4* could cause defects in DNA repair and sensitize cancer cells to apoptosis. PAWR was found to be upregulated only in response to apoptosis but not to other processes such as growth arrest or necrosis. Because of its association with apoptosis, *PAWR* is regarded as a tumor inhibitor (25). In CRC, SRC inhibitor and 5-FU could promote PAWR induced apoptosis and responses to treatment (26).

Immunotherapy has revolutionized cancer treatment and reinvigorated the field of tumor immunology. Immune cells, especially T cells, can be harnessed to eliminate tumor cells (27). In general, increases in infiltrating CD8 T cells have been associated with longer OS (28). In the present study, however, enrichment of CD8 T cells was greater in patients in cluster C2, who have a poorer prognosis, than in cluster C1. Key covariates, like tumor progression, should also be considered, as higher densities of CD8 T cells were associated with more advanced tumors. Macrophages are tissue-resident differentiated monocytes, traditionally divided into M1 and M2 subtypes according to their differentiation status and function (29). M1 macrophages have pro-inflammatory activities, enhancing anti-tumor TH1 response, whereas M2 macrophages are anti-inflammatory, favoring the establishment of a tolerogenic microenvironment (30). Consistent with previous findings, the present study found that M2 macrophage densities were higher in cluster C2 than in cluster C1. DCs are essential professional antigen-presenting cells that direct T cell activation and differentiation (31). Elevated densities of CD208+ mature DCs have been associated with poor prognosis in patients with CRC (32). Similarly, activated DCs were more enriched in cluster C2. Immune infiltrates in the TME have been found to contribute to tumor growth and progression, as well as the prognoses of patients with CRC (33). In contrast, the present study showed that patients in the senescence cluster C2, characterized by TME activation, had higher SRG risk scores than patients in the senescence cluster C1, characterized by TME inhibition. An increase in tumor stage has been associated with reductions in immune cell density and the immune core, suggesting that immunotherapy might benefit CRC patients with high-risk SRG scores.

Variations in gut microbiota, including bacteria, viruses, and fungi, have been associated with many pathologic conditions, including various cancers, intestinal bowel diseases (IBDs), hepatic steatosis, type 2 diabetes, and obesity (34). Changes in the gut microbiome enhance environmental risk, resulting in the initiation and enhancement of CRC (35). Alterations in the gut microbiome have been observed during relatively early phases of colorectal carcinogenesis and have been employed to identify individuals at risk for colorectal adenoma, the precursor lesion to CRC (36). In the present study, *Bacteroidia* were more enriched in cluster C2 patients, suggesting that *Bacteroidia* enrichment was associated with poor prognosis. Similar, infiltration of the enterotoxigenic *Bacteroides fragiles* (ETBF), a member of the class *Bacteroidia*, was found to be significantly higher in the tumor than in the adjacent normal tissues (37). ETBF has been shown to induce tumorigenesis in azoxymethane/dextran sodium sulfate (AOM/DSS) induced colitis-related murine models of CRC (38). ETBF positivity was found to be more frequent in patients with sporadic premalignant lesions (39) and familial adenomatous polyposis (40) than in their respective controls. Metagenomic analysis has shown that *Bacteroides fragilis* is the only species consistently enriched in the gut microbiomes of patients with CRC worldwide (41).

In conclusion, the present study showed that senescence-related subtypes and a signature consisting of eight SRGs were associated with CRC patient prognosis and clinicopathological features, as well as with immune cell infiltration and gut microbiota. These findings may enable better prediction of CRC patient prognosis and facilitate individualized treatments.

## Data Availability Statement

The datasets presented in this study can be found in online repositories. The names of the repository/repositories and accession number(s) can be found in the article/Supplementary Material.

## Author Contributions

L-CY and Y-FP contributed to conception and design of the study. J-JD, Y-YF, X-QZ, WC, M-FY and X-HC analyzed the data. J-JD was a major contributor in writing the manuscript. All authors read and approved the final manuscript.

## Funding

This work was supported by the National Natural Science Foundation of China (Nos. 81572291 and 82172816) and Zhejiang Provincial Natural Science Foundation of China (Nos. LY19H160022 and Y22H162686).

## Conflict of Interest

The authors declare that the research was conducted in the absence of any commercial or financial relationships that could be construed as a potential conflict of interest.

## Publisher's Note

All claims expressed in this article are solely those of the authors and do not necessarily represent those of their affiliated organizations, or those of the publisher, the editors and the reviewers. Any product that may be evaluated in this article, or claim that may be made by its manufacturer, is not guaranteed or endorsed by the publisher.
