# Supplementary material for: Identification of Senescence-Related Subtypes, the Development of a Prognosis Model, and Characterization of Immune Infiltration and Gut Microbiota in Colorectal Cancer
Source: Front Med (Lausanne). 2022 May 26;9:916565. doi: 10.3389/fmed.2022.916565 (PMC9198838; doi:10.3389/fmed.2022.916565)
Supplement: Supplementary file 4 [file Table_4.DOCX]

Supplementary Material


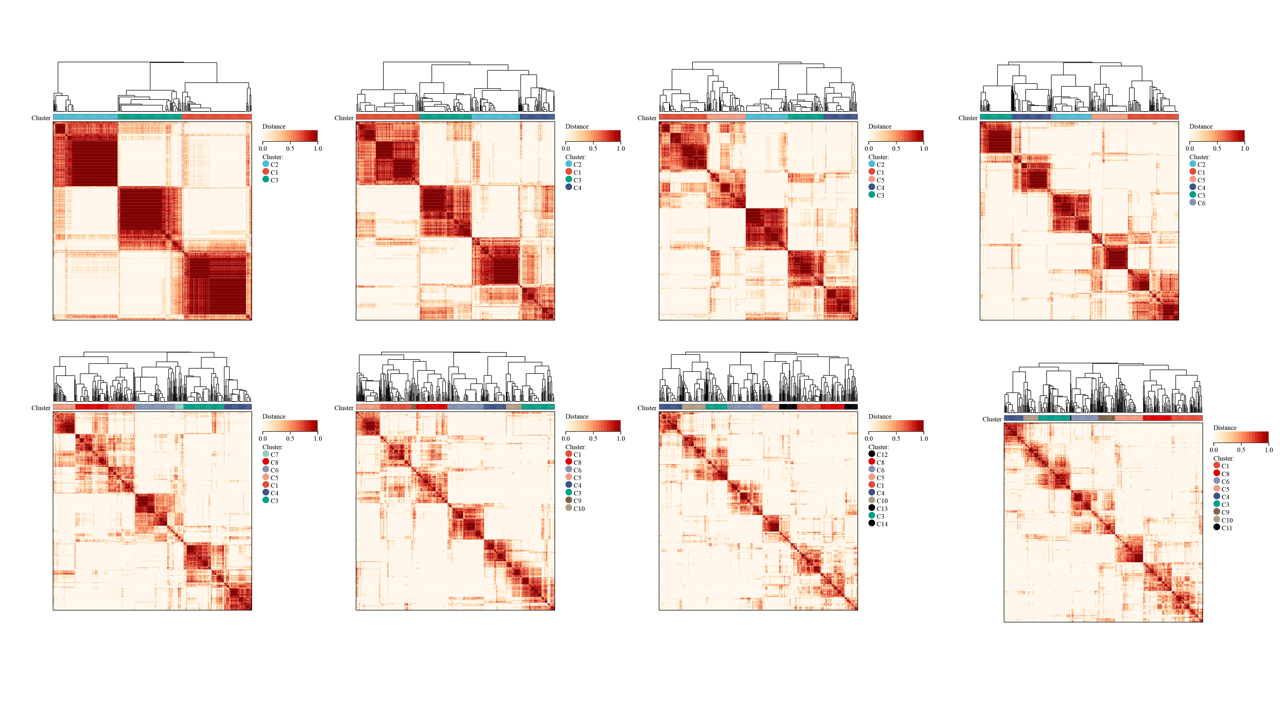


**Supplementary Figure 1.** Unsupervised clustering of SRGs and consensus matrix heatmaps for k = 3-9.


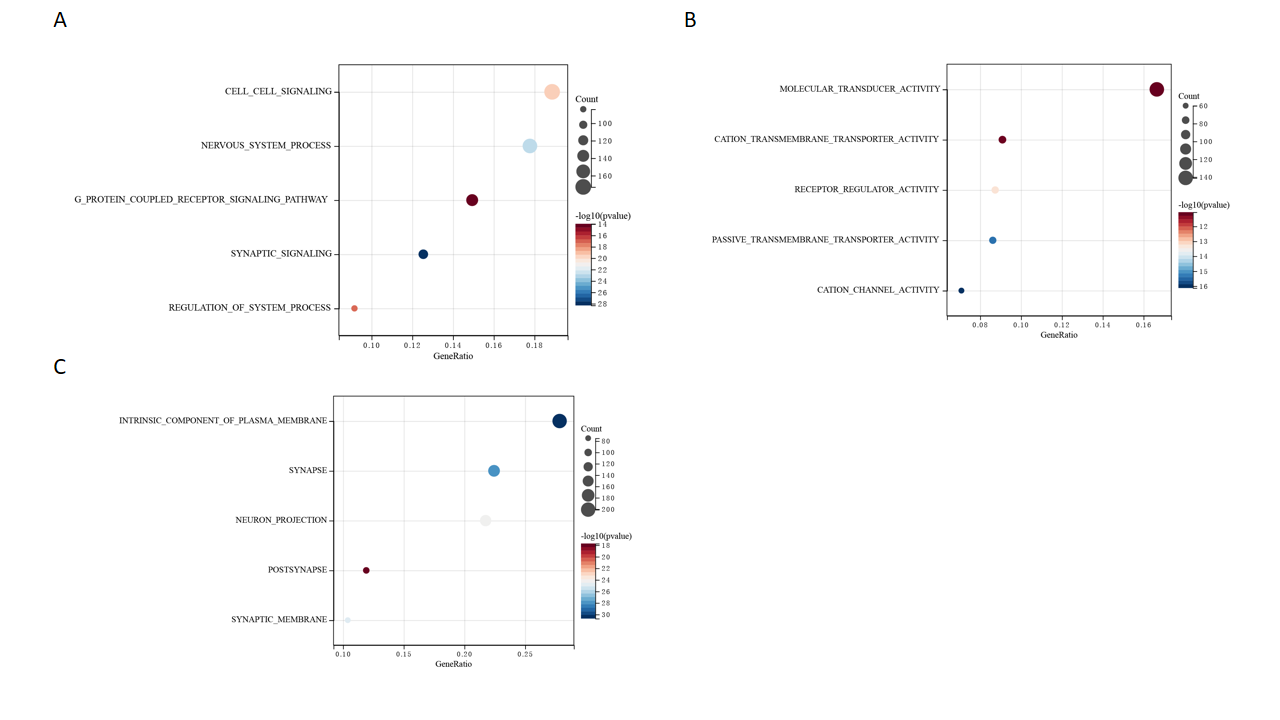


**Supplementary Figure 2.** GO enrichment analyses of DEGs in the two subtypes.
